# Income composition inequality of Chinese residents and fiscal redistribution effect: An empirical analysis on individual income tax and transfer system

**DOI:** 10.1371/journal.pone.0296129

**Published:** 2024-01-02

**Authors:** Xunhua Tu, Jie Yan, Jing Zheng

**Affiliations:** 1 School of Public Finance and Taxation, Southwestern University of Finance and Economics, Chengdu, Sichuan, China; 2 Department of Economics & Management, Sichuan Engineering Technical College, Deyang, Sichuan, China; Bucharest University of Economic Studies: Academia de Studii Economice din Bucuresti, ROMANIA

## Abstract

Based on the data of China Family Panel Studies (CFPS), this paper decomposed Chinese residents’ income into labor income and capital income by income source, and measured the income inequality and income composition inequality of Chinese residents during 2010–2018. We take the Gini coefficient as a measure of inequality and, by decomposing it by income source, analyze the absolute and relative marginal effects of capital income and labor income on the overall income inequality. On this basis, this paper discusses the redistributive effect of financial instruments such as personal income tax and transfer payment on income inequality and income composition inequality. The results show that capital income is not only the main driving factor for the increase of overall income inequality, but also its influence on inequality is gradually increasing. The results of the redistribution effect of fiscal instruments show that although individual income tax and transfer payment both help to reduce the overall income inequality, only individual income tax can reduce the inequality of income composition, while transfer payment will exacerbate it. In the background of the rising share of capital income, it may widen the income distribution gap in the long run. Hence, future fiscal redistribution efforts should consider the income composition inequality. This includes further promotion of individual income tax reforms, optimization of the tax rate structure, enhancement of relevant tax laws governing capital income like property income, and continuous improvement in the redistributive impact of fiscal instruments.

## Introduction

In recent years, the share of capital income has increased while the share of labor income has decreased. The dynamic change of factor income share and its potential impact on individual income inequality have attracted more and more attention. The study of dynamic changes in factor shares can not only establish a link between income at the macroeconomic level (national accounts) and income at the household level, but it can also help to understand individual income distribution inequality and the relationship between social justice and equity of different income sources [1]. Since 1999, China’s labor-income share has been steadily declining, falling from 59.33% in 1999 to 52.06% in 2008 before slowly rebounding, but still fluctuating. From 2010 to 2021, the composition of China’s income changed dramatically, with property income increasing from 6.2% to 8.8%, with an average growth rate of 15.1%. The average growth rate of property income is 15.1%, which is significantly higher than the 9.7% growth rate of wage income. Ranaldi (2019) [2] refers to income composition inequality as the extent to which different income components, such as capital and labor, are unevenly distributed in income distribution. The analysis of income composition inequality reflects the changing balance of power between capital income share and labor income share in the primary distribution result, which is the combination of income element distribution and individual income distribution result. Inequality research from a multiple perspective, such as income composition, can help clarify redistributive preferences and identify specific policy tools to address the growing gap between rich and poor, the decline in the political weight of labor, and the rise in inequality [3].

The level of labor income share can reflect how much the fruits of economic development are shared by workers and is an important indicator of the degree of common prosperity [4]. China has always placed a high value on raising workers’ wages, closing the income distribution gap, and striving for common prosperity, and fiscal tools such as personal tax and transfer payments, as important means of income redistribution, play an important role in regulating the income distribution gap and promoting common prosperity.The Outline of the 14th Five-Year Plan for National Economic and Social Development and Vision 2035 of the People’s Republic of China emphasizes not only increasing the proportion of labor remuneration in the initial distribution, improving the policy system of factor distribution, and increasing the property income of urban and rural residents through multiple channels, but also clearly emphasizes the importance of fiscal redistribution. The Central Finance and Economics Committee’s tenth meeting emphasized the need to "adjust the high, expand the middle, and raise the low" by raising taxes, social security contributions, and transfer payments, creating an olive-shaped distribution structure, fostering social justice, and enabling everyone to make significant strides toward the objective of shared prosperity. The report of the 20th CPC National Congress emphasizes the need to improve the distribution system, increase the income of low-income earners, expand the middle-income group, regulate the order of income distribution, and standardize the mechanism of wealth accumulation. There have also been numerous studies on the redistributive effects of fiscal tools such as personal taxes and transfer payments, with the general conclusion that the income regulation effect of fiscal tools is weak and inefficient. In a context characterized by high income inequality among residents and significant disparities in income composition, exploring the redistributive effects of fiscal tools from the dual perspectives of income inequality and income composition can provide valuable insights. This approach not only aids in gaining a deeper understanding of the composition of income inequality but also helps clarify the relationship between initial income distribution outcomes and fiscal redistribution. It allows for a more comprehensive assessment of fiscal redistribution effects, thereby facilitating a more effective utilization of fiscal tools in promoting shared prosperity and offering useful insights towards achieving a more equitable society.

This study assesses the distribution of total income, labor income, and capital income among income subgroups and dissects the Gini coefficient by income sources to investigate their respective contributions to income inequality. Utilizing the CFPS 2018 data for calculation, our analysis reveals that capital income, including property, is predominantly concentrated among the top earners. In 2018, the highest 5% of income earners received 63.36% of all capital income. Notably, the capital income Gini coefficient reached a remarkably high value of 0.9647. Moreover, the increasing share of capital income exacerbates personal income inequality, with a 1% rise in capital income corresponding to a 0.0385% increase in the total income Gini coefficient. The study measures the redistributive impacts of fiscal instruments, such as personal taxes and transfer payments, on income inequality and income composition inequality in China. The findings reveal that while both personal taxes and transfer payments contribute to alleviating overall income inequality, personal taxes specifically address income composition inequality in structural terms. In contrast, transfer payments exacerbate income composition inequality and may potentially worsen income inequality over the long term.

This paper’s possible marginal contributions are primarily three: First, it broadens the research scope of the fiscal redistribution effect. The existing literature mostly evaluates the fiscal redistribution effect from the perspective of income inequality, but this paper starts from the inequality of residents’ income composition, uses the factor concentration curve to reflect the degree of inequality of residents’ income composition in China, and then uses the data to empirically analyze the redistribution effect of fiscal tools such as personal tax and transfer payments on income composition inequality; Second, it decomposes the Gini coefficient by income source and combines income inequality and income composition inequality for analysis, enriching and improving the relevant literature in the fields of income inequality and fiscal redistribution; Third, it compares and analyses the redistributive effects of fiscal tools on income inequality and income composition inequality, providing micro evidence for further optimizing the redistributive effects of fiscal tools.

The remainder of the paper is organized as follows: in the literature review part, a brief review of the research literature on related topics is provided; in the methods and data section, we introduce the calculation method and data used in this paper; in the analysis section, we analyze the results of income inequality and income composition inequality in China; and in the empirical analysis section, we empirically examined the fiscal redistribution effect; and finally, the paper concludes with research findings and pertinent recommendations.

## Literature review

This section will sort out the relevant studies from three perspectives, including factor distribution and income inequality, income composition and fiscal redistribution, and the method to measure the effect of fiscal redistribution.

### Factor distribution and income inequality

According to Franzini and Pianta (2015) [5], the factor distribution of income is a direct indicator of the power balance between labor and capital. The impact of factor distribution on individual incomes is significant, as labor and capital remunerations received by individuals or households are expressed as labor income and capital income, respectively. The role of factor shares in historical studies of income inequality should be considered along with other indicators [6]. **On the one hand, a high capital income share can increase interpersonal income inequality.**Rising capital income shares are often cited as one of the causes of increasing interpersonal income inequality. Bilbiie et al. (2022) [7] show that when capital and income inequality coexist, the two have complementary effects. Capital income tends to be more underestimated than labor income due to measurement error and conceptual differences, weakening the impact of capital shares and their dynamics on income inequality [8]. Fräßdorf et al. (2011) [9] use a comparative analysis of cross-country data to find that although the share of capital income is low, its contribution to overall inequality is proportionally higher (about twice the share of capital income in total income). Piketty (2014) [10] contends that due to the typically higher inequality in capital income compared to labor income, a reduction in the share of labor income and an increase in the share of capital income would intensify income inequality. Milanovic (2017) [11] decomposes the total Gini coefficient by the source of income to reveal the relationship between capital share and personal income inequality. The rise in the share of capital income transmits to three conditions for rising personal income inequality: high savings rate for capital income, high concentration of assets, and high correlation between capital income ranking and total income ranking. **On the other hand, the higher share of capital income among the top earners further exacerbates income inequality.** Bengtsson and Waldenstrom (2018) [12] empirically find that in the long run, the share of capital income is strongly correlated with the income of the top earners, and there is an almost one-to-one relationship between the growth of the two. This suggests a dominance of capital income among top earners. Atkinson and Lakner (2021) [13] find that capital income shares and labor income shares have become increasingly linked since the 1980s in the United States, with the top 1% of earners almost always belonging to the top 20% of richest capitalists. Burdín et al. (2022) [14] analyze the evolution of the top income group and capital income shares in Uruguay based on social security, personal and corporate income tax records, and household survey microdata and find that inequality in the tail of the data is increasing and is driven primarily by the rising share of capital income. Sarkar and Tuomala (2021) [15] find that as asset markets expand, the share of income flowing to the top cohort increases, and asset price growth is significantly and positively associated with income inequality at the top. **Third, the measure of the impact of factor distribution on income inequality.** Atkinson and Bourguignon (2000), Atkinson (2009) [1, 16] measure the link between factor distribution and income inequality by decomposing the square of the coefficient of variation of income, showing the conditions under which an increase in the income share of capital translates into an increase in overall income inequality, and measuring the income standard deviation. Francese and Mulas-Granados (2015) [17] decompose the Gini coefficient by the source of income and find that if the capital share is high enough, the labor income share increases contribute to lower inequality and vice versa. Atkinson and Lakner (2021) [13] propose another measure of the impact of factor distribution on income inequality by using a rank-based association measure to reflect the relationship between labor and capital income. Ranaldi (2019) [2] decomposes total income into two capital and labor income factors, defines income composition inequality as the extent to which income composition is unequally distributed in the income distribution, and constructs an index measuring income composition inequality to measure the strength of the link between factor distribution and income inequality.

### Income composition inequality and fiscal redistribution

Ranaldi (2022) [18] presents a theoretical analysis regarding the impact of fiscal redistribution instruments such as taxes and transfers on income composition inequality, with a focus on the context of rising capital income shares. The study finds that in the long run, such instruments aimed at reducing income inequality may increase income composition inequality. Iacono and Palagi (2020) [19] provide empirical evidence on the effects of double income tax reforms (DITRs) implemented in the Nordic countries in the early 1990s. The study finds that DITRs, by lowering the marginal tax rate on capital income, contributed to capital income concentration and higher concentration at the top, which led to an increase in income composition inequality. Petrova and Ranaldi (2021) [3] argue that taxation and redistribution policies can have significant effects on income composition inequality. Specifically, they argue that tax reforms favoring capital income may shift capital income to the top of the income distribution, resulting in a more unequal distribution of income composition. Moreover, redistributive welfare policies aimed at low- and middle-income households, and higher social spending can increase the concentration of labor income at the bottom of the distribution, leading to an increase in income composition inequality. Zhan and Yu (2022) [20] examine the effect of government subsidies on labor income share and find an inverted U-shaped relationship. In other words, government subsidies can significantly increase labor income share when they are below a critical value, but excessive subsidies can trigger an increase in rent-seeking costs of firms and inhibit the growth of labor income share.

### The method to measure the effect of fiscal redistribution

Numerous scholars have investigated the factors influencing the impact of fiscal redistribution and its adjustments on income disparities. Yue et al. (2021) [21] highlighted that previous literature commonly used changes in residents’ income gaps before and after fiscal redistribution to gauge the direction and magnitude of the redistribution effect. Specifically, some scholars commence by assessing the overall redistribution effect and employ various methods to break it down into distinct components. For instance, Kakwani (1984)、Čok (2013) [22, 23] decomposed the redistributive effect into horizontal equity and vertical equity, revealing both the progressive and horizontal inequality effects. Other scholars focused on measuring the marginal redistribution effect. For example, Lerman and Yitzhaki (1985) [24] along with Lu et al. (2018) [25] decomposed the Gini coefficient based on income sources and evaluated the marginal change in income inequality contributed by different income components, thereby measuring the marginal effect of redistribution. Additionally, Urban (2014) [26] proposed the marginal effect decomposition method to measure the contribution of fiscal instruments to vertical and horizontal impacts.

### Some other factors that affect the inequality

The choice of inequality measurement method significantly affects the outcomes, potentially leading to different conclusions. For example, Cowell and Flachaire (2023) [27] noted that the traditional Gini coefficient tends to underestimate income inequality, and the Gini inequality index is sensitive to the inclusion or exclusion of the highest income earners [28]. Additionally, inflation plays a substantial role in income distribution. Sintos (2023) [29] discovered that factors such as price stability, financial deepening, development level, state employment, and fiscal redistribution enhance income equality in a given country. Furthermore, low inflation strengthens the income-equalizing effect of fiscal redistribution. Chletsos and Sintos (2023) [30] proposed that the overall impact of financial development on income inequality averages to zero but varies systematically based on study characteristics. In contrast, Chletsos and Sintos (2022) [31] found that IMF programs are associated with increased income inequality for up to five years. Heimberger (2020) [32] utilized a new dataset with 1254 observations from 123 primary studies, employing meta-analysis and meta‐regression techniques, and discovered that financial globalization has a more significant and markedly stronger impact on increasing inequality. Finally, Li and Liu (2019) [33] provided overall support for a small negative relationship between financial liberalization and income inequality, considering potential publication bias and method heterogeneity.

A comprehensive review of the literature reveals that income inequality and fiscal redistribution have been studied more thoroughly in the literature, but the focus has primarily been on studying changes in capital share and labor share from a macro perspective or changes in labor income share and capital share from an enterprise perspective, and there is a dearth of literature that discusses the effects of fiscal redistribution from the perspective of individual income structure in conjunction. Starting with individual income and dividing it into capital income and labor income helps to link the distribution of initial factor-based distribution with the distribution results of individual income inequality, and discussing the redistributive effect of fiscal tools on this basis can provide a more comprehensive understanding of the redistributive effect of fiscal tools. Considering the current huge differences in the composition of residents’ income in China, incorporating the perspective of residents’ income composition is essential when delving into the redistribution of income via fiscal instruments. Ranaldi’s (2022) [18] article is the most similar to this paper in that it examines the impact of fiscal redistribution on income composition inequality primarily from a theoretical standpoint. Based on the article, this paper investigates the redistributive effects of fiscal instruments on income inequality and resident income composition inequality empirically. Given that the share of capital income is increasing, we attempt to make some useful additions to income distribution studies by investigating the effects of fiscal instruments on income composition inequality, as well as providing some new ideas and references for the selection of variables to measure the effectiveness of fiscal redistribution.

## Methods and data

### Method to calculate income composition inequality

According to Ranaldi (2019) [2], the degree of uneven distribution of various income components (such as capital and labor) in income distribution is referred to as income composition inequality. Contrarily, inequality in income composition is minimized when each person has the same percentage of income composition. Income composition disparity is highest when individual income at the top and bottom of the income distribution originates from two different categories. The degree to which changes in factor shares and overall income disparity are related can be seen in income composition inequality. Ranaldi (2019) [2] creatively creates the IFC (Income Factor Concentration) index to measure income composition disparity, building on the idea of the Gini coefficient metric, whose fundamental logic is as follows:

- The study decomposes the sample income into two sources, capital income and labor income.
- Using this decomposition, Lorenz curves of income are constructed based on the ascending order of individual income. Subsequently, the concentration curves of capital and labor are calculated separately. The Lorenz curve of total income is then obtained by summing the concentration curves of capital and labor at each point.
- Furthermore, the study constructs the zero and maximum concentration curves based on the minimum and maximum inequality of income composition.
- Finally, the income concentration index (IFC) is calculated to measure the degree of inequality of income composition.

The formula of income concentration index (IFC) is:

$$\text{IFC}=\frac{A(z)}{B^{\text{max}}(z)}$$ (1)

**Fig 1:**
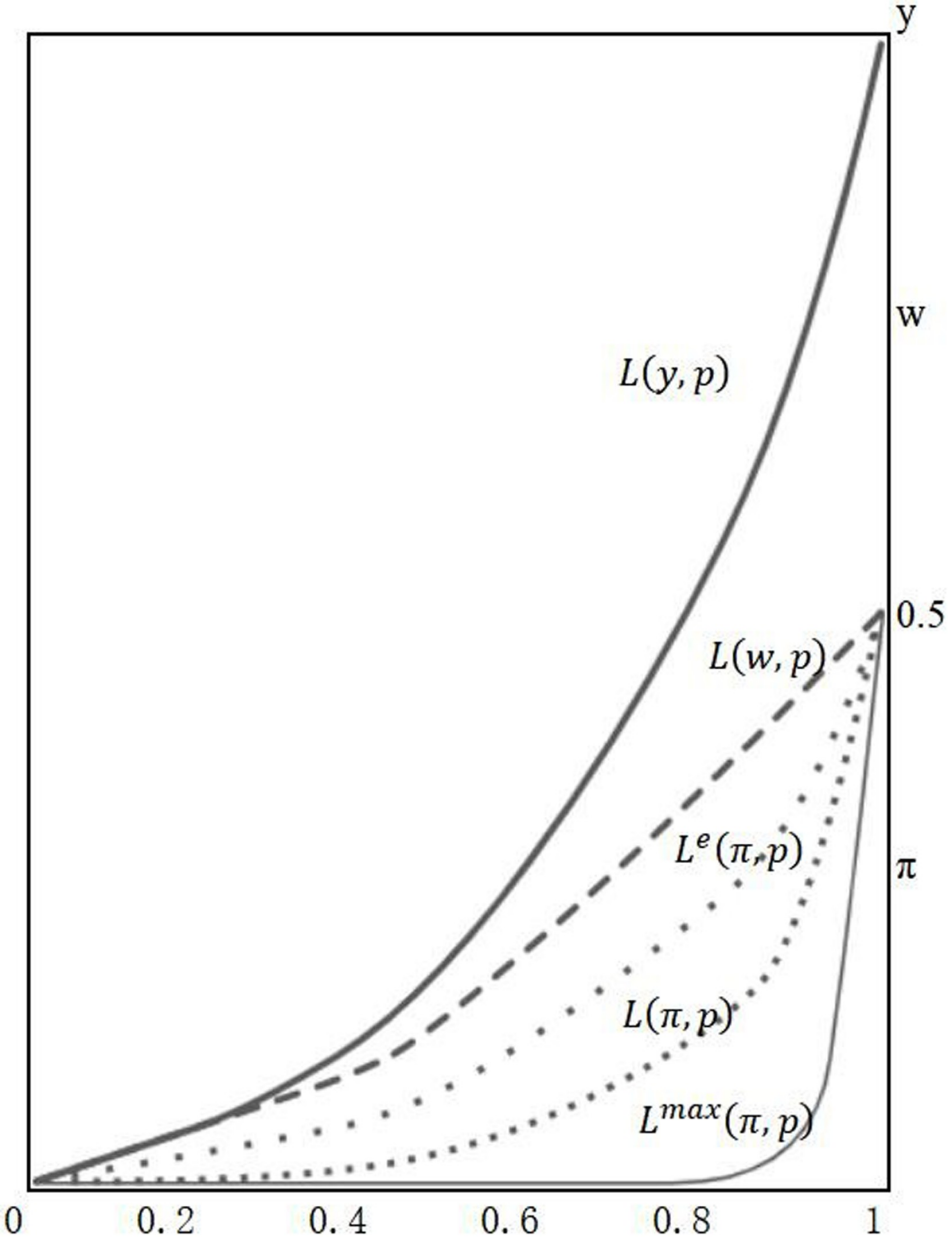
Concentration curve of income factors. Note: In the depicted figure, the x-axis indicates the population distribution across quantiles, while the y-axis shows the income distribution. The Lorenz curve for income is denoted by L(y, p), whereas L(w, p) and L(π, p) represent the concentration curves for labor and capital income, respectively. Moreover, L^e^(π, p) stands for the zero concentration curve of capital income, which reflects perfectly equal income composition (π = w = 1/2), whereas L^max^(π, p) represents the maximum concentration curve of capital income under maximally unequal income composition.

The variable z represents the source of income (either capital income π or labor income w). A(z) denotes the area between the zero concentration curve of the income source z, denoted as L^e^(z, p), and the actual concentration curve, denoted as L(z,p). Similarly, B^max^ (z) represents the area between the zero concentration curve L^e^(z, p) and the maximum concentration curve L^max^(z, p) for the income source z. Fig 1 illustrates the Lorenz curve of income, concentration curves of income factors, as well as the approximate shapes of the zero and maximum concentration curves. The measurement method used in this paper is borrowed from this approach to estimate income inequality in China.

Moreover, the concentration indices for capital income and labor income can be written separately:

$$\text{Capital}\;\text{income}\;\text{concentration}\;\text{index}:\;IFC_{\pi }=\frac{\omega \pi \left({\tilde{\mu }}_{\omega }-{\tilde{\mu }}_{\pi }\right)}{B_{\pi }}$$ (2)

$$\text{Labor}\;\text{income}\;\text{concentration}\;\text{index}:\;IFC_{\omega }=\frac{\omega \pi \left({\tilde{\mu }}_{\pi }-{\tilde{\mu }}_{\omega }\right)}{B_{\omega }}$$ (3)

In formula (2), ${\tilde{\mu }}_{\pi }=\frac{1}{2n}\overset{}{\underset{}{\sum_{i=0}^{n}\left(\sum_{j=0}^{i}\alpha_{j}+\sum_{j=0}^{i+1}\alpha_{j}\right)}}\;,\alpha_{j}$ denotes the ratio of capital income to total capital income for individual j. In the same way, ${\tilde{\mu }}_{\omega }=\frac{1}{2n}\overset{}{\underset{}{\sum_{i=0}^{n}\left(\sum_{j=0}^{i}\beta_{j}+\sum_{j=0}^{i+1}\beta_{j}\right)}}\;,$ and *β*_*j*_ is the ratio of labor income to total capital income for individual j. ${\tilde{\mu }}_{\pi }$ and ${\tilde{\mu }}_{\omega }$ denote the areas of the concentration curves of capital and labor, respectively. Combining Eqs (2) and (3) with Fig 1, it can be seen that when Eq (2) is positive, the area of ${\tilde{\mu }}_{\omega }$ is greater than *${\tilde{\mu }}_{\pi }$* which indicates that capital income is more concentrated in the top income group and labor income is concentrated at the bottom, while the opposite is true when Eq (3) is positive, and *IFC*_*π*_ = -*IFC*_*ω*_.

### Method to calculate income inequality

To provide a more intuitive reflection on the role of different income components in contributing to income inequality, this study employs the decomposition method proposed by Lerman and Yitzhaki (1985), López-Feldman (2006), and Lu et al. (2018) [24, 25, 34] to disaggregate the Gini coefficient. This approach factors in the contribution of each income source to the overall Gini coefficient as the product of three components: the Gini coefficient of the income source itself (G_K_), the share of income in total income (S_K_), and the correlation between the income source and the ranking of total income (R_k_). The calculation formula for this decomposition is specified as follows:

$$\text{Gini}=\sum_{\text{k}=1}^{\text{K}}\left[\frac{\mathrm{cov}\left({\text{y}}_{\text{k}},\text{F}\right)}{\mathrm{cov}\left({\text{y}}_{\text{k}},{\text{F}}_{\text{k}}\right)}\right]\;\times \;\left[\frac{2\mathrm{cov}\left({\text{y}}_{\text{k}},{\text{F}}_{\text{k}}\right)}{{\text{m}}_{\text{k}}}\right]\;\times \;\left[\frac{{\text{m}}_{\text{k}}}{\text{m}}\right]=\sum_{\text{k}=1}^{\text{K}}{\text{R}}_{\text{k}}{\text{G}}_{\text{k}}{\text{S}}_{\text{k}}$$ (4)

Specifically, cov(y_k_, F) represents the covariance between the cumulative distribution of income source k and income, while m and m_k_ denote the average income and average income of income category k, respectively. The correlation between the ranking of income source k and total income, denoted as R_k_, is a critical factor in the decomposition process and ranges from -1 to 1, depending on the nature of the relationship between income category k and total income. A value of 1 indicates a monotonically increasing relationship, while a value of -1 reflects a monotonically decreasing relationship. In the case where income source k is constant, the value of R_k_ is 0. G_k_ represents the Gini coefficient of income source k, while S_k_ denotes the share of income source k in total income.

Eq (4) provides a framework for investigating the impact of changes in individual income sources on overall income inequality. Specifically, by holding all other income sources constant and increasing the income of source k by a factor of (1+e_k_) as e_k_ approaches zero, we can estimate the marginal change in the Gini coefficient (Q).


$$\text{Q}=\frac{\partial G}{\partial e_{k}}=S_{k}\left({\text{R}}_{\text{k}}{\text{G}}_{\text{k}}-\text{G}\right)$$ (5)

Dividing both sides of Eq (5) by G at the same time, we get

$$\frac{Q}{G}=\frac{S_{k}{\text{R}}_{\text{k}}{\text{G}}_{\text{k}}}{G}-{\text{S}}_{\text{k}}$$ (6)

Eq (6) is employed to estimate the marginal effect of income source k on the total Gini coefficient, expressed as $\frac{Q}{G}$, which reflects the percentage change in the total Gini coefficient due to a change in income category k. Additionally, $\frac{S_{k}{\text{R}}_{\text{k}}{\text{G}}_{\text{k}}}{G}$ represents the ratio of the inequality contribution of income source k to the total Gini coefficient. S_k_ is the share of income source k in total income. To carry out this analysis, we divide the sample into labor income and capital income categories and decompose the Gini coefficient by income source. We also investigate the redistributive effects of financial instruments such as personal taxes and transfer payments. Notably, the sum of the relative marginal effects of each income category is zero, highlighting the importance of examining the impact of each income source in a comprehensive manner.

### Methods for evaluating the effects of fiscal redistribution

The present study contributes to the literature on fiscal redistribution by examining the marginal changes in the Gini coefficient resulting from personal taxes and transfer payments. The methodology employed draws on the work of Lerman and Yitzhaki (1985), López-Feldman (2006), and Lu et al. (2018) [24, 25, 34], who decompose the Gini coefficient by income source to assess the marginal contribution of personal taxes and transfers to income inequality. The method and procedure to measure the redistributive effect of personal income tax and transfer payments on income composition inequality are similar, differing only in the substitution of the Gini coefficient, representing income inequality, with the IFC index, reflecting income composition inequality.

To measure the redistributive effect of personal taxes, individual pre-tax income is inverted based on the tax rates and comprehensive deductions in the revised 2018 individual income tax law using data from the CFPS survey. The Gini coefficient of pre-tax market income decomposition is then calculated separately by income source, capital income and labor income, and compared with the Gini coefficient measured by the decomposition of after-tax income (excluding transfer payments). This analysis examines the effect of personal taxes on regulating income from different income sources and analyzes the change in the difference between the contribution of capital income and labor income to inequality.

To measure the redistributive effect of transfer payments, the Gini coefficient is decomposed using market income before personal taxes, divided into capital income, labor income, and transfer income by income source, and then compared with the Gini coefficient measured using the decomposition of income added to transfer payments but not subject to personal taxes. This analysis reflects the redistributive effect of transfer payments on income inequality.

Finally, to measure the combined redistributive effects of personal taxes and transfer payments, the Gini coefficient is first measured by decomposing the disposable income of individuals after taxes and including government transfers, and then compared with the Gini coefficient measured by decomposing the income before taxes without transfers. This analysis reflects the redistributive effects of personal taxes and transfers and shows the changes in the contribution of capital income and labor income to inequality before and after personal taxes and transfers.

### Data for calculating income composition inequality and income inequality

The primary data source for this study is the China Family Panel Studies (CFPS), which provides comprehensive income data at the individual, household, and community levels. The national sample survey data spans from 2010 to 2018 and covers 25 provinces/municipalities/autonomous regions, offering a broad representation of the population and the ability to track dynamic changes in individual and household income over time. In terms of the sampling procedure, CFPS utilizes an implicit stratification method to create a multi-stage probability sample. The sample selection occurs in three stages. The first stage (PSU) involves selecting administrative districts or counties, the second stage (SSU) involves choosing administrative villages or neighborhood committees, and the third stage (end-stage or TSU) involves households. The first two stages of sampling rely on official administrative division data, while the third stage employs a map-based approach to construct the end-stage sampling frame. Household samples are drawn using a random start and systematic equal-interval sampling method. By utilizing this rich dataset, the study aims to provide a robust analysis of income composition and its impact on overall income inequality.

1. Household income. The CFPS survey statistics measure total (net) household income as the sum of five income components, namely, wage income, total (net) business income, property income, transfer income, and other income. These income components are further disaggregated according to their sources. Wage income is defined as after-tax wages, bonuses, and benefits in kind earned by family members engaged in non-farm work or agriculture. Business income represents the net income after deducting costs incurred from agriculture, forestry, animal husbandry, sideline, and fishery production, the value of agricultural products produced by the family for consumption, and the net profit earned by the family from self-employment or private enterprise. Transfer income is the income households receive through government transfers such as pensions, grants, and relief, as well as social contributions. Property income refers to the income derived from renting out land, houses, production materials, and other property. Other income comprises financial support and gifts from relatives and friends, gratuities, and other sources. To assess the concentration of capital and labor factors, the present study categorizes household income into labor and capital income, including property income, interest, dividends, and other related income sources.

The CFPS survey data only includes the principal and market value of stocks and funds, the stocks and funds were most likely not purchased in the same year, and the dividends and fund income components are difficult to estimate. The definition of final labor income encompasses wage income, other income (as transfer payments from relatives and friends), and government transfer payments. As operating income is a result of both labor and capital, this study adopts Iacono and Ranaldi’s (2021) [35] approach to identify the capital income portion as the operating income exceeding the average wage and the labor income portion as the operating income below or equal to the average wage. In investigating the impact of fiscal redistribution on income composition inequality, this paper compares income composition inequality with and without transfers. This paper excludes samples with negative capital and labor income and zero total income.

2. Household equivalence population size. To address potential disparities in family population sizes and age distributions, this study draws on the practice of Kou et al. (2021) [36], uses the number of adults in a family as the equivalent number of adults to calculate the per capita income of the family, and calculates the income composition inequality index of Chinese residents accordingly.

Table 1 lists the descriptive statistics of the main variables from 2010 to 2018.

**Table 1:** Descriptive statistics for main data yuan.

Year/Sample size	Variable name	Mean	Standard deviation	Min	Max
2010 (N = 14511)	Total income	10501.75	30794.88	0.6	3002794
	Labor income	7845.72	12774.53	0	522603.9
	Capital income	695.43	25840.7	0	2977396
	Transfer income	1960.599	5484.07	0	133333.3
2012 (N = 13255)	Total income	12016.33	21779.54	0.17	1518023
	Labor income	9031.25	19876.07	0	1518023
	Capital income	721.002	4579.956	0	254610.8
	Transfer income	2432.86	6732.29	0	189640
2014 (N = 13255)	Total income	15521.49	20748.11	0.2	980000
	Labor income	11402.16	17572.56	0	980000
	Capital income	1071.85	6612.815	0	255900.1
	Transfer income	3047.476	8592.24	0	500000
2016 (N = 13668)	Total income	19416.27	37342.41	0.45	1949235
	Labor income	13357.52	19004.84	0	745788.3
	Capital income	2252.1	20980.81	0	1768635
	Transfer income	3806.65	12038.66	0	500000
2018 (N = 14030)	Total income	25740.15	60620.09	0.5	5660000
	Labor income	19073.88	28870.65	0	1333333
	Capital income	2135.92	48326.46	0	5449201
	Transfer income	4530.348	12955.02	0	600000

Note:Collated and calculated according to CFPS household economy database

### Data for calculating fiscal redistribution effects

1. Income data. Primarily utilizing statistics from the 2018 China Family Panel Studies (CFPS) survey, this study employs the aforementioned analytical method to process income data, decomposing it into capital income and labor income.
2. Personal tax data. The present study utilizes income data from the 2018 China Family Panel Studies (CFPS) survey to obtain pre-tax income through inverse extrapolation in conjunction with the relevant provisions of the tax law. In 2018, China adjusted and revised the personal income tax law and started to implement it on January 1, 2019. Specifically, the study extrapolates individual pre-tax income based on the reformed individual income tax law in 2018. The process involves three main steps. First, the study determines the number of social security contributions that individuals need to pay, including basic pension insurance, medical insurance, unemployment insurance, and housing fund. The data for these contributions are obtained from the individual contribution amounts of basic social security and housing provident fund for individuals in the CFPS 2018 survey database. Second, the study identifies special additional deductions for children’s education, continuing education, major medical care, housing loan interest, housing rent, and support for the elderly by combining the household demographic characteristics, medical care and education level, and mortgage in the database. Finally, the study combines the tax rates in the new tax law to invert the pre-tax income based on the classified after-tax income.
3. Transfer payments. The present study categorizes transfers into two types: government transfers and transfers between residents’ relatives and friends. Government transfers are primarily comprised of the total government grants recorded in the CFPS database. Meanwhile, transfers between friends and relatives are mainly reflected in the other income recorded in the CFPS database.

## Analysis of income inequality and income composition inequality among Chinese residents

This section first calculates the total income share, capital income share, and labor income share of Chinese people grouped by income and then makes a preliminary determination of the changes in the income composition of various groups according to their trends using the income data from the CFPS2010–2018. The trend in income inequality and income composition inequality among Chinese residential households is then estimated using the Gini coefficient of total income and the factor concentration index, and the relationship between the two is tentatively inferred from the trend. To account for the impact of residents’ income composition on income inequality, the Gini coefficient is finally decomposed into capital income inequality and labor income inequality.

### Trends in the overall income shares and inequality

To accurately represent the income distribution from the lowest to the highest, the sample is divided into five strata based on income percentiles: 0–10%, 10–50%, 50–90%, 90–95%, and 95–100%. An assessment of the association between income composition and income inequality is conducted through the examination of the share of different income categories within these clusters, the income composition inequality index, and the Gini coefficient during the period of 2010 to 2018. The trends of total income shares, overall income inequality, and income composition inequality for each income cluster are presented in Table 2.

**Table 2:** Income shares, Gini coefficient, and IFC for different income groups %.

Income grouping	2010	2012	2014	2016	2018
0–10	0.75	0.43	0.43	0.44	0.45
10–50	15.53	16.18	15.64	14.8	14.71
50–90	45.03	48.32	45.88	43.51	44.38
90–95	11.95	11.59	11.05	10.96	11.59
95–100	26.74	23.47	27.00	30.29	28.87
Gini	0.4984	0.4759	0.4988	0.5364	0.5635
IFC	0.7783	0.3376	0.5698	0.6170	0.6564

Note: Self-measured based on CFPS database household economic roll data.

Table 2 presents three salient features that characterize the distribution of income in China. First, the concentration of income towards the top is evident, with the share of income held by the bottom 0–10% income cohort declining from 0.75% in 2010 to 0.43% in 2012 and stabilizing at a low level of approximately 0.4% thereafter. The income share of households in the bottom 10%-50% also exhibits a downward trend, decreasing from 15.53% in 2010 to 14.71% in 2018. In contrast, the income share of households in the top 5% cohort increases, with the share rising from 26.74% in 2010 to 28.87% in 2018 and peaking at 30.29% in 2016. This trend is indicative of income concentration at the top. Secondly, the Gini coefficient rose from 0.4984 in 2010 to 0.5635 in 2018, with a pattern consistent with that of the income share of the top 5% group, indicating that the concentration of income towards the top contributes to widening income gaps and intensifying income inequality. Thirdly, income composition inequality displays a "V-shaped" pattern, with the income composition inequality index (IFC) reaching 0.7783 in 2010, declining to 0.3376 in 2012, and subsequently increasing to 0.6564. The significant drop in the IFC index in 2012 may be attributed to the 2012 tax reform in China, known as "replace business tax with value-added tax," which markedly reduced corporate profits [37]. This reduction, in turn, led to a decrease in the proportion of capital income among residents, aligning with the observed decline in the capital income share of the top 5% group in Table 4.

### Trends in the labor income shares

Table 3 presents changes in labor income shares across different income groupings from 2010 to 2018, and the observed trends are consistent with those of total income shares. Specifically, the labor income share of the bottom 0–10% cohort has remained at a very low level since its decline in 2012, accounting for only around 0.4%. The labor income share of the 10%-50% cohort experienced a slight decrease, declining from 16.23% in 2010 to 15.62% in 2018. The share of labor income of the 50%-95% cohort exhibited minor fluctuations, reaching a peak of 49.08% in 2012 and a trough of 45% in 2016. The labor income share of the 90%-95% cohort declined from 12.27% in 2010 to 11.46% in 2018. In contrast, the top 5% cohort saw an increase in labor income share, rising by 2.4 percentage points from 23.87% in 2010 to 26.25% in 2018. These findings suggest that labor income shares are also concentrated towards the top.

**Table 3:** Share of labor income of different income groups %.

Income grouping	2010	2012	2014	2016	2018
0–10	0.78	0.43	0.45	0.46	0.47
10–50	16.23	16.65	16.56	15.66	15.62
50–90	46.85	49.08	46.96	45.00	46.20
90–95	12.27	11.62	10.91	10.76	11.46
95–100	23.87	22.21	25.13	28.12	26.25

Note: Self-measured based on CFPS database household economic roll data

### Trends in the capital income shares

Table 4 in this study provides information on the capital income shares of different income groups, and four main data features can be observed. Firstly, the capital income shares of the bottom 0–10% and 10%-50% income groups have consistently remained at low levels, with the former group’s share always below 0.5%. Secondly, the capital income share of the 50%-90% income group exhibits an inverted "V" trend, gradually shifting towards the top 90%-95% income group, which reached its peak at 34.72% in 2012 and declined to 20.46% in 2018. Thirdly, the top 90%-95% income group’s share of capital income has slowly increased over time, from 6.36% in 2010 to 13.28% in 2018. Fourthly, the top 5% income group’s share of capital income has remained consistently high, yet with significant volatility and a "V"-shaped trend. It reached its highest point of 77.23% in 2010, declined to 45.97% in 2012, and then gradually rebounded to 63.36% in 2018.

**Table 4:** Share of capital income for different income groups %.

Income grouping	2010	2012	2014	2016	2018
0–10	0.25	0.46	0.16	0.18	0.22
10–50	3.13	7.82	3.49	2.8	2.67
50–90	13.02	34.72	31.50	22.58	20.46
90–95	6.36	11.04	12.97	13.64	13.28
95–100	77.23	45.97	51.88	60.80	63.36

Note: Self-measured based on CFPS database household economic roll data

**Fig 2:**
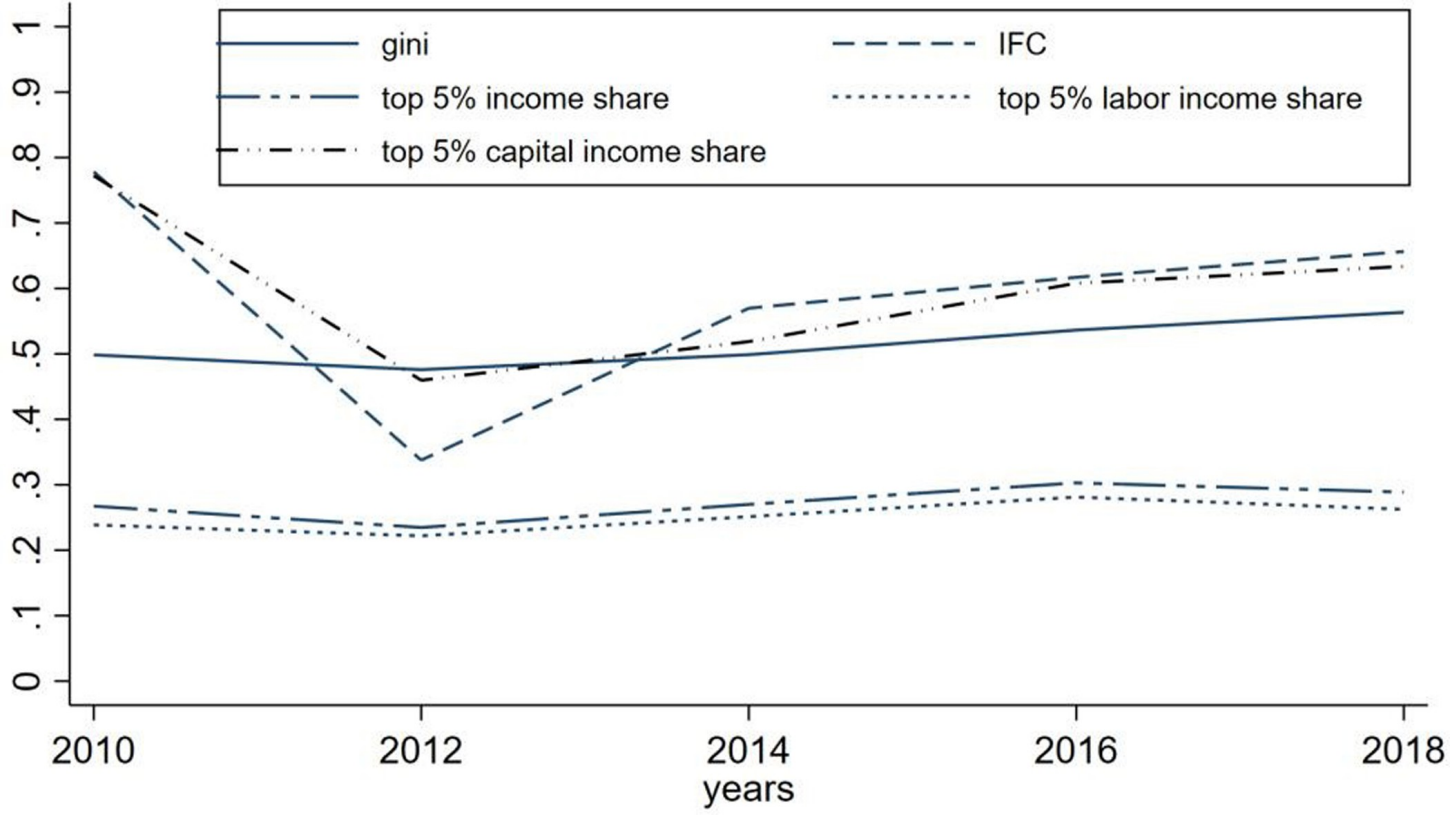
The share of the top 5% of income groups and the trend of the IFC index and Gini coefficient.

Overall, the data reveals a concentration of income among the top 5% group, resulting in higher income inequality. The labor share and total income share follow a similar trend, while capital income share is concentrated in the top income group, with the lowest and highest shares being 45.97% and 77.23%, respectively. This study finds a positive relationship between the concentration of capital income in the top group and income composition inequality, leading to an exacerbation of overall income inequality. Fig 2 provides a visual representation of this relationship, highlighting the impact of income composition imbalance on income inequality.

The findings of this study are presented in Fig 2, which plots the income share of the top 5%, labor income share, and capital income share against the IFC index and Gini coefficient. The results demonstrate that the trend of the capital income share of the top 5% aligns with the trend of the IFC index, indicating that income composition inequality is closely associated with the concentration of capital in the top group. Moreover, while the Gini coefficient exhibits a smaller change, its trend is similar to that of the IFC index, suggesting that changes in income composition inequality are positively related to income inequality. Furthermore, the trend of the labor income share and total income share is comparable, implying that changes in the labor income share may not be the primary cause of changes in income composition inequality.

### The contribution of the income composition to income inequality

As delineated in the previous section, the Gini coefficient decomposition technique has been applied to analyze the distribution of household income during the period of 2010–2018. Specifically, this approach involves decomposing the Gini correlation between capital income and labor income, and total income (Rc, Rl), as well as determining the Gini coefficients of capital income and labor income (Gc, Gl), along with the respective shares of capital income and labor income (Sc or Sl) utilizing equation (4). The findings of this analysis have been presented in Table 5.

**Table 5:** Income inequality disaggregated by source of income.

Breakdown of projects	2010	2012	2014	2016	2018
Capital income correlation (Rc)	0.8897	0.7099	0.8069	0.8702	0.8634
Gini coefficient of capital income (Gc)	0.978	0.9285	0.9515	0.9383	0.9647
Share of capital income (Sc)	0.0539	0.055	0.0703	0.0681	0.0709
Inequality share of capital income (Ic)	0.0903	0.0712	0.1037	0.102	0.1094
Relative income inequality (Ic/Sc)	1.6753	1.2945	1.4751	1.4978	1.543
Relative marginal impact (Ic-Sc)	0.0364	0.0162	0.0334	0.0339	0.0385
Labor income correlation (Rl)	0.9969	0.987	0.9828	0.9937	0.9892
Gini coefficient of labor income (Gl)	0.5008	0.5069	0.5105	0.5287	0.523
Share of labor income (Sl)	0.9461	0.945	0.9297	0.9319	0.9291
Share of Labor Income Inequality (Il)	0.9097	0.9287	0.8963	0.898	0.8908
Relative income inequality (Il/Sl)	0.9615	0.9828	0.9641	0.9636	0.9588
Relative Marginal Impact (Il-Sl)	-0.0364	-0.0163	-0.0334	-0.0339	-0.0383
Total Gini coefficient (Gini)	0.5192	0.5091	0.5204	0.5452	0.5396

Note: The share of capital income inequality (Ic) and labor income inequality (Il) are expressed as a ratio of capital income and labor income inequality to the Gini coefficient (Gini) multiplied by the respective income share (Sc or Sl) as given by the formula *I*_*c*_ = *R*_*c*_ **×** *G*_*c*_ **×** *S*_*c*_/*Gini*. The relative income inequality (Ic/Sc) represents the ratio of the share of capital income inequality (Ic) to the share of capital income (Sc). The relative marginal impact (Ic-Sc) refers to the difference between the share of capital inequality (Ic) and the share of capital income (Sc). The aforementioned outcomes have been measured based on the household equivalent size-adjusted disposable income per capita, and the results are presented in Tables 5 and 6.

**Table 6:** Results of measuring fiscal redistribution effects on income inequality.

Redistribution Tools	Breakdown of income components	R(k)	G(k)	S(k)	I(k)	I(k)/S(k)	I(k)—S(k)
Income before personal taxes	Capital Income	0.7856	0.9665	0.085	0.0993	1.1682	0.0143
	Labor income	0.9888	0.6468	0.915	0.9007	0.9844	-0.0143
	Total income before taxes		0.6497				
Personal income after tax	Capital Income	0.7521	0.9645	0.0697	0.0791	1.1349	0.0094
	Labor income	0.99	0.6389	0.9303	0.9209	0.9899	-0.0094
	Total after-tax income		0.639				
Transfer payments (before personal taxes)	Capital Income	0.8072	0.9665	0.0731	0.0909	1.2435	0.0178
	Labor income	0.9588	0.6468	0.7868	0.7778	0.9886	-0.009
	Government Transfers	0.6776	0.9012	0.1127	0.1097	0.9734	-0.003
	Inter-resident transfers	0.5229	0.9408	0.0273	0.0214	0.7839	-0.0059
	Total revenue		0.6273				
Transfer payments (after personal taxes)	Capital Income	0.7791	0.9645	0.059	0.0719	1.2186	0.0129
	Labor income	0.9566	0.6389	0.7884	0.7817	0.9915	-0.0067
	Government Transfers	0.6828	0.9012	0.1228	0.1226	0.9984	-0.0002
	Inter-resident transfers	0.5222	0.9408	0.0297	0.0237	0.798	-0.006
	Total revenue		0.6166				

Note: R(k) represents the correlation between income category k and total income inequality, G(k) represents the Gini coefficient of income category k, S(k) indicates the share of income category k in total income, I(k) denotes the share of income inequality category k in total income inequality, I(k)/S(k) measures the degree of relative inequality of income category k, and I(k)-S(k) measures the relative marginal impact on income inequality.

Table 5 presents the income inequality decomposition analysis by income source, allowing for a clearer comparison of the contribution of different income sources to overall income inequality. The results reveal several key findings. First, there is a lower correlation between capital income and total income inequality (Rc), while labor income is more strongly related to income inequality, with a correlation that remains at a high level above 0.98 from 2010 to 2018 (Rl). Second, the impact of capital income on overall income inequality is relatively low, with capital income share (Sc) remaining at a low level and mainly concentrated in the top income group, while the share of labor income (Sl) has declined slightly but remains high above 0.92. Third, the degree of capital income inequality (Gc) is much higher than that of labor income inequality (Gl), with capital income being highly differentiated and mainly concentrated among a small group of people. In contrast, labor income inequality has shown a slow upward trend, but it is still lower than capital income inequality and overall income inequality. Fourth, the relative income inequality of capital income (Ic/Sc) is higher than that of labor income (Il/Sl), indicating that the marginal growth of capital income has a relatively larger impact on income inequality. The relative marginal impact of capital income on total income inequality (Ic-Sc) is positive, indicating that the high inequality level of capital income mainly drives the growth of the Gini coefficient. Conversely, the relative marginal impact of labor income on total income inequality (Il-Sl) is negative, indicating that growth in labor income helps to reduce income inequality. These findings shed light on the importance of different income sources in driving income inequality, with labor income playing a more significant role than capital income.

## Empirical analysis of fiscal redistribution effect

Drawing upon the decomposition analysis of the Gini coefficient by income sources in China, this study integrates redistributive mechanisms, specifically personal taxes and transfer payments, into the analytical framework to evaluate their impact on the inequality of residents’ income and income composition inequality.

### The fiscal redistribution effects on income inequality

Drawing upon the methodology of decomposing the Gini coefficient by income source, as previously proposed, the current study evaluates the redistributive effects of personal taxes and transfer payments on income inequality. Using a redistributive effect evaluation framework, the findings are presented in Table 6.

The redistributive effects of personal tax, transfer payments, and the combined effect of personal tax and transfer payments were derived from the results presented in Table 6. Firstly, the redistribution effect of personal tax was measured by comparing the decomposition results of the Gini coefficient of personal income before and after personal tax. The results indicate that personal tax alleviates both total income inequality and disaggregated income inequality, with a reduction of 0.0107 in total income inequality and a decrease of 0.002 and 0.0079 in the Gini coefficients of capital income and labor income, respectively. Furthermore, the personal tax reduces the effect of capital income inequality on total income inequality by decreasing the correlation between capital income and total income inequality and the share of capital income inequality in total income inequality. However, it is worth noting that capital income inequality remains high after personal taxes, indicating that personal tax has a limited ability to regulate income inequality.

Secondly, to evaluate the redistributive influence of transfer payments, we can contrast the Gini coefficients computed using individual pre-tax total income with and without transfer payments, effectively isolating the impact of transfers and excluding the influence of personal taxes. The results indicate that, First, transfer payments reduce income inequality, but they do not regulate capital income inequality. After adding transfer income, total income inequality is reduced by 0.0224. The Gini coefficient of capital income did not change before and after the transfer. Second, transfer payments exacerbate the impact of capital income inequality on total income inequality. After adding transfer income, the correlation between capital income and gross income inequality increases by 0.0216, and the share of capital income and the share of capital income inequality both decrease, but the relative marginal impact of capital income changes on gross income inequality increases by 0.0035, and the increase of capital income by 1% will increase the Gini coefficient of gross income by 0.0178%. Third, the marginal redistribution effect of government transfer payments and inter-resident transfer payments. A 1% increase in income from these two transfers reduces the Gini coefficient of total income by 0.003% and 0.0059%, respectively. The transfer payment between residents plays a greater role in redistribution, which may be due to the Chinese tradition of "helping" and relief between relatives and friends, which plays a certain role in redistribution, and this part of transfer payment income also flows more to low-income households.

Thirdly, the combined redistributive effect of personal tax and transfer payments was derived by comparing the decomposition results of the Gini coefficient of total income after personal tax and transfer payments and income before personal tax. The results indicate that both personal tax and transfer payments significantly reduce income inequality and disaggregated income inequality while mitigating the effect of capital income inequality on total income inequality. However, the high level of capital income inequality remains after the implementation of personal tax and transfer payments, implying that their ability to regulate income inequality is limited. Overall, the redistributive effect of transfer payments on income inequality is greater than that of personal tax, while the combined redistributive effect of personal tax and transfer payments is the strongest and has the greatest alleviation of income inequality. In terms of disaggregated income inequality, personal tax can effectively alleviate capital income and labor income inequality, while transfer payments cannot regulate capital income inequality and exacerbate the positive effect of capital income inequality on total income inequality.

### The fiscal redistribution effects on income composition inequality

Drawing on Petrova and Ranaldi (2021) [3] and existing literature, the preceding section of this paper divides residents’ income into two categories: capital income and labor income. The transfer payments are included in labor income while analyzing the redistributive effects. Eq (1) is used to calculate the IFC index for income before personal taxes, income after personal taxes, income before personal taxes plus transfers, and income after personal taxes plus transfers. The results of the analysis are presented in Table 7, which demonstrates the effects of personal taxes and transfers on income composition inequality.

**Table 7:** Results of measuring fiscal redistribution effects on income composition inequality.

Revenue caliber	Income before personal taxes	Personal income after tax	Income before personal taxes plus transfer payments	After-tax income plus transfer payments
IFC	0.3164	0.2451	0.4122	0.3537
Gini	0.6497	0.639	0.6272	0.6166
ΔIFC	0	-0.0713	0.0958	0.0373
Δgini	0	-0.0107	-0.0225	-0.0331

Note: The change in both ΔIFC and ΔGini refers to the difference between the value of the corresponding income column and the value obtained from the calculation of income before taxes.

The findings presented in Table 7 indicate that personal taxes have a significant effect in reducing income composition inequality and income inequality. Specifically, the post-tax IFC index is 0.0713 lower and the Gini coefficient is 0.0107 lower compared to pre-tax. On the other hand, transfer payments have a stronger moderating effect on total income inequality but exacerbate income composition inequality. Prior studies have noted that transfer payments are unable to regulate capital income inequality and mostly target low-income individuals with labor income, resulting in a 0.0225 decrease in the Gini coefficient but a 0.0958 increase in income composition inequality. This influence of transfer payments can be attributed to the assessment of inequality in income composition, which examines the distribution of two distinct income types among different segments of the population. As transfers are considered a part of labor income, their effect is primarily felt by the lower end of the income spectrum. Therefore, when transfers are increased, they further concentrate income towards the bottom, leaving the distribution of capital income unaffected. This results in a greater disparity in income composition. The combined effect of personal taxes and transfer payments on total income inequality is the strongest, but it also leads to an increase in income composition inequality. The Gini coefficient decreases by 0.0331 and the IFC index increases by 0.0331 due to the combined moderating effect of personal taxes and transfer payments. However, the increase in income composition inequality caused by transfer payments offsets the moderating effect of personal taxes on it.

## Conclusions and recommendations

The impact of income composition on inequality, as well as the effects of personal taxes and transfer payments on income composition inequality and long-term income inequality, are of critical importance. This study uses data from the CFPS survey to analyze and measure the income composition inequality index of Chinese residents and decompose the Gini coefficient by income source to evaluate the redistributive effects of personal taxes and transfer payments on income inequality and income composition inequality.

The paper finds that income shares are concentrated at the top and substantial inequality are evident in the income composition of Chinese residents, with the income share of the top 5% group increasing from 26.74% in 2010 to 28.87% in 2018. The share of labor income for the top 5% group increased from 23.87% in 2010 to 26.25% in 2018, while the share of capital income remained high, peaking at 77.23% in 2010 and falling to 45.97% in 2012 before rebounding to 63.36% in 2018. In terms of inequality, the Gini coefficient rose from 0.4984 in 2010 to 0.5635 in 2018, with income concentration at the top exacerbating income inequality and the income composition inequality index (IFC) at 0.7783 in 2010, rising to 0.6564 in 2018 after dropping to 0.3376 in 2012.

Furthermore, the study shows that capital income has a high contribution to income inequality, with changes in capital income being positively correlated with total income inequality. Although the share of capital income in total income is low, its inequality is high. The marginal increase in capital income has a greater relative impact on inequality than labor income. Additionally, redistribution policies such as personal taxes and transfer payments are effective in regulating the income distribution gap and reducing income inequality. While both personal taxes and transfer payments help alleviate income inequality, the redistributive effect of transfer payments on income inequality is greater. The redistributive effect of personal taxes plus transfer payments is the strongest and has the greatest alleviation of income inequality. However, while personal tax can effectively alleviate the inequality of capital income and labor income, transfer payments cannot regulate the inequality of capital income and exacerbate the positive effect of capital income inequality on total income inequality.

Finally, the study finds that there are differences in the effects of redistributive policies such as personal taxes and transfer payments on regulating inequality in income composition. Individual taxes reduce income composition inequality, while transfer payments widen income composition inequality. The IFC index measured using after-tax income decreases by 0.0713 compared to pre-tax, and transfer payments increase income composition inequality by 0.0958. Overall, the findings suggest that income composition has a significant impact on inequality and that personal taxes and transfer payments can be effective tools in regulating income distribution and reducing income inequality in China.

The income distribution in China is characterized by significant disparities, with capital income being predominantly concentrated among the top 5% of residents. This trend is expected to further exacerbate income polarization and widen the income distribution gap. To address this issue, this paper offers a set of policy recommendations. Firstly, it is crucial to broaden the sources of income for middle and low-income groups, particularly in terms of property income. Despite the significant growth of property income, it is still largely concentrated among high-income groups. To increase property income for the middle and low-income groups, investment channels should be broadened, rural collective property rights systems should be reformed, and rural assets should be revitalized. Additionally, rural specialty industries should be developed to create a more stable income space for rural residents.

Secondly, personal income tax reform should be pursued to improve the regulatory capacity of the income distribution. At present, the income composition of Chinese residents varies significantly, with capital income accounting for a large proportion of high-income groups, while labor income mainly pertains to the middle and low-income groups. Despite being progressive, individual income tax’s effectiveness in regulating income disparity is limited, as the marginal tax rate of labor income is high, and that of non-labor income is even lower. To address this issue, the highest marginal tax rate of labor income should be lowered, and the structure of the individual income tax rate should be optimized. The amount of deduction for tax should also be adjusted differently, with a greater deduction for mortgage interest to increase the disposable income of middle and low-income groups. Finally, tax laws related to capital income, such as property income, should be improved to enhance individual income tax’s ability to regulate income distribution.

Thirdly, the accuracy of transfer payments should be improved, with a focus on "raising the low" of transfer payments. Transfer payments should be targeted at low-income groups, with increased coverage and levels of low-income assistance. Transfer payments should also be increased to deal with unexpected shocks such as unemployment assistance and other social assistance expenditures to increase residents’ ability to fight against risks and maintain a stable life. The structure of transfer payments should be optimized by raising the level of rural pension benefits and narrowing the gap between urban and rural groups’ pension benefits.

Lastly, income inequality should be a crucial variable in measuring the effectiveness of redistributive policies. The fiscal redistribution policy aimed at reducing income inequality should focus on the part of income that mainly causes income inequality, i.e., income source Z. Thus, the IFC index should be a key variable in measuring effective redistribution policies.

## Supporting information

S1 Dataset (DTA)
